# Elacridar Inhibits BCRP Protein Activity in 2D and 3D Cell Culture Models of Ovarian Cancer and Re-Sensitizes Cells to Cytotoxic Drugs

**DOI:** 10.3390/ijms26125800

**Published:** 2025-06-17

**Authors:** Piotr Stasiak, Justyna Sopel, Artur Płóciennik, Oliwia Musielak, Julia Maria Lipowicz, Agnieszka Anna Rawłuszko-Wieczorek, Karolina Sterzyńska, Jan Korbecki, Radosław Januchowski

**Affiliations:** 1 Institute of Biological Sciences, Faculty of Exact and Natural Sciences, University of Zielona Góra, 65-417 Zielona Góra, Poland; p.stasiak@cm.uz.zgora.pl (P.S.); 113690@g.elearn.uz.zgora.pl (O.M.); 2 The Doctoral School of Exact and Technical Sciences, University of Zielona Góra, 65-417 Zielona Góra, Poland; 3 Institute of Health Sciences, Collegium Medicum, University of Zielona Góra, 65-417 Zielona Góra, Poland; j.sopel@inz.uz.zgora.pl (J.S.); a.plociennik@inz.uz.zgora.pl (A.P.); j.korbecki@inz.uz.zgora.pl (J.K.); 4 Department of Histology and Embryology, Doctoral School, Poznan University of Medical Sciences, 61-701 Poznań, Poland; julia.lipowicz@student.ump.edu.pl; 5 Department of Histology and Embryology, Poznan University of Medical Sciences, 61-701 Poznań, Poland; arawluszko@ump.edu.pl (A.A.R.-W.); k.olejniczak@ump.edu.pl (K.S.)

**Keywords:** ABC transporters activity, breast cancer resistance protein (BCRP), chemotherapy resistance, elacridar, molecular inhibitors, multidrug resistance (MDR), ovarian cancer, three-dimensional (3D) cell culture

## Abstract

Chemotherapy resistance is a major obstacle in the treatment of ovarian cancer, often resulting in disease recurrence and poor prognosis for patients. A key contributor to this resistance is the overexpression of ATP-binding cassette (ABC) transporters, including breast cancer resistance protein (BCRP/ABCG2), which actively effluxes chemotherapeutic agents such as topotecan (TOP) or mitoxantrone (MIT), limiting their intracellular accumulation and efficacy. This study investigated the potential of elacridar (GG918), a potent dual P-gp and BCRP inhibitor, to overcome drug resistance in ovarian cancer cell lines. Both TOP-sensitive and TOP-resistant ovarian cancer cells were grown in two-dimensional (2D) monolayers and three-dimensional (3D) spheroid models to better mimic the tumor microenvironment. The expression of the ABCG2 gene was quantified via qPCR and BCRP protein levels were assessed by western blotting and immunofluorescence. Drug response was evaluated using MTT viability assays, while BCRP transporter activity was examined using flow cytometry and microscopic assessment of the intracellular retention of BCRP fluorescent substrates (Hoechst 33342 and MIT). In both 2D and 3D cultures, elacridar effectively inhibited BCRP function and significantly enhanced sensitivity to TOP. These findings suggest that elacridar can inhibit BCRP-mediated drug resistance in ovarian cancer cell models.

## 1. Introduction

Ovarian cancer represents a considerable challenge in gynecologic oncology. It is a heterogeneous cancer characterized by a variable clinical course, ranking eighth in the world among cancer deaths in women [1]. In contrast, it ranks third in terms of the incidence of gynecologic cancers [1]. The high mortality observed in ovarian cancer is due to the diagnosis of the disease at an advanced stage (stage III, IV according to the FIGO classification) and the development of resistance to treatment [2]. Accounting for 90% of primary ovarian malignancies, epithelial ovarian cancer (EOC) is divided into two types, differing in patterns of tumor progression and genetic mutations present [2,3]. Low-grade neoplasms are referred to as type I tumors. They arise from endometriosis or borderline tumors, including low-grade serous carcinomas, low-grade endometrioid carcinomas, mucinous carcinomas, and certain types of clear cell carcinomas. In contrast, tumors with an elevated degree of malignancy are classified as type II tumors. These tumors have unidentified precursors and include high-grade serous and endometrioid carcinomas, undifferentiated carcinomas, and some clear cell carcinomas and carcinosarcomas [2].

The typical care for EOC consists of surgical removal of the tumor, followed by chemotherapy treatment [4]. First-line chemotherapy includes the use of cisplatin (CIS) or carboplatin in combination with paclitaxel (PAC) [4]. Despite the use of surgery and appropriate first-line chemotherapy, 5% of patients are primary resistant to treatment and 70–80% of patients with EOC develop resistance and relapse [5] after varying lengths of treatment [6,7].

When the response to first-line chemotherapy is assessed, patients are defined as platinum-sensitive, partially platinum-sensitive, platinum-insensitive, or platinum-resistant. Depending on the specific platinum-sensitivity group, a second-line chemotherapy strategy is selected accordingly. In platinum-responsive patients, CIS/carboplatin and taxanes are used in second-line chemotherapy. In patients not responding to platinum, second-line chemotherapy is composed of topotecan (TOP), liposomal doxorubicin (DOX), or gemcitabine [4,7]. Unfortunately, the response to second-line chemotherapy is normally around 10–15%, due to the development of drug resistance [4].

DOX and TOP are the most important drugs used in second-line ovarian cancer chemotherapy. DOX belongs to the inhibitors of DNA topoisomerase II, an enzyme responsible for introducing supercoils in the DNA molecule after replication [8]. The drug mainly acts after replication to form irreversible covalent cross-links between topoisomerase and DNA, causing breakage of the DNA and subsequent death of the cell [9]. TOP, a semisynthetic camptothecin derivative, is an alkaloid extracted from the *Camptotheca acuminate* tree [10]. It is utilized to treat small cell lung cancer (SCLC) [11], ovarian cancer [12], and cervical cancer [13]. It inhibits DNA topoisomerase I, an enzyme that regulates over- or under-winding of the DNA helix [14,15]. Stabilization of the enzyme–DNA complex by TOP leads to the inhibition of DNA replication and transcription, resulting in cancer cell death. Cells in the S-phase of the cell cycle are most vulnerable to TOP action [16,17].

Different mechanisms of resistance to TOP can be present in cancer cells. Mutations in topoisomerases or a decrease in the expression of these enzymes make them less sensitive to TOP [18]. Nevertheless, the most crucial mechanism of TOP resistance is the active drug removal from cancer cells by the ABC family drug transporters [19]. The key transporter that actively removes TOP from cancer cells is the breast cancer resistance protein (BCRP), encoded by the *ABCG2* gene [20]. This gene has been overexpressed in breast cancer and ovarian cancer tumors and cell lines [20,21,22]. Resistance to TOP may also result from overexpression of a different gene, *ABCB1* (*MDR1*), which encodes P-glycoprotein (P-gp) [22,23,24,25].

Both P-gp and BCRP are among the members of the ABC protein family of drug transporters [20,26]. These proteins use the energy obtained from ATP hydrolysis to actively remove cytotoxic agents from the cell [27]. Overexpression of ABC family proteins results in the development of multidrug resistance (MDR), which is the most important mechanism of resistance at the cellular level. MDR is a phenomenon in which chemotherapy-treated cancer cells acquire cross-resistance to many different drugs, regardless of their diverse chemical structure, leading to the ineffectiveness of further treatment [28].

The *BCRP* gene was originally identified in 1998, when it was cloned from MCF-7/AdrVp, a multidrug-resistant breast cancer cell line [29]. BCRP protein expression was associated with the treatment of cancer cells with chemotherapeutics, such as mitoxantrone (MIT) and TOP [29,30]. The *ABCG2* gene is located on chromosome 4q22 [29,30] and is composed of 16 exons [31]. BCRP is a polytopic, transmembrane protein (TM) containing 655 amino acids. It has one nucleotide-binding domain (NBD), one membrane-spanning domain (MSD), and 6 TM α-helices [30]. The molecular mass of BCRP is about 60 kDa, and it increases to about 70 kDa after N-glycosylation at position Asn596. BCRP is a “half-transporter,” and homodimerization is essential to achieve the activity of BCRP [32,33].

Under physiological conditions, BCRP is strongly expressed on the apical membranes of placental syncytiotrophoblasts, intestinal epithelium, liver hepatocytes, brain microvascular endothelial cells, and renal proximal tubular cells [30]. BCRP has also been shown to be strongly expressed in the mammary gland of mice, cows, and humans, especially during lactation, resulting in the secretion of clinically and toxicologically important substances, including drugs, toxins, and vitamins, into milk [29,34]. Under physiological conditions, it contributes to the absorption, distribution, and elimination of drugs and endogenous compounds and it takes part in guarding tissues from exposure to xenobiotics and endogenous toxins [29,30]. BCRP can control the cellular homeostasis of physiologically important endogenous compounds, like heme, porphyrins, riboflavin, or estrogen [29]. It has about 200 substrates. These include chemotherapeutics such as camptothecin derivatives, MIT, methotrexate, TOP, flavopiridol, and irinotecan and its active analog, SN-38; inhibitors of tyrosine kinase: imatinib, gefitinib, and nilotinib; non-chemotherapeutic drugs: prazosin, nitrofurantoin, sulfasalazine, and rosuvastatin; and non-therapeutic compounds, including dietary flavonoids, porphyrins, the lipid phosphatidylserine, uric acid, vitamins, estrone 3-sulfate (E1S), and the carcinogen 2-amino-1-methyl-6-phenylimidazo[4,5-b]pyridine (PhIP) [29,30]. Nucleoside and nucleotide analogues, like AZT and lamivudine, are also substrates of BCRP [29,30]. Accordingly, the FDA has identified BCRP as a key drug transporter that plays a significant role in the distribution of various therapeutics [30].

Unfortunately, high expression of BCRP protein is prevalent in many other cancers. Elevated expression of this protein has been observed in breast [35], ovarian [21], brain [36], liver [36], and lung cancer [36], as well as in acute myeloid leukemia (AML) [36]. The elevated expression levels of this protein were noted in cancer stem cells, which have implications for cancer treatment [36]. We reported the overexpression of BCRP protein in five TOP-resistant ovarian cancer cell lines [22,24].

The very high number of substrates and expression in many cancers makes BCRP an important factor in cancer resistance to chemotherapy and other treatments. Thus, therapeutic strategies that can inhibit the activity of these proteins are under investigation.

During decades of investigation, different classes of ABC protein inhibitors have been developed. In general, ABC protein inhibitors are divided into first generation, second generation, and third generation inhibitors, differing in specificity and induced side effects [37]. First-generation inhibitors are characterized by low specificity; moreover, they usually interact with other proteins, which can lead to various side effects. Second-generation inhibitors have higher specificity through modifications made to the molecule. Third-generation inhibitors, on the other hand, are made to optimally inhibit the target protein [38]. The use of ABC family protein inhibitors, in combination with cytotoxic drugs, is expected to result in the maintenance of adequate chemotherapeutic concentrations in cancer cells, which is made possible by the inhibition of ABC proteins. Such synergistic action is expected to ensure an adequate therapeutic effect [39,40].

Elacridar, the object of our research, is a third-generation inhibitor [41]. It is known under many names, such as GG918, GF120918, or N-{4-[2-(1,2,3,4-tetrahydro-6,7-dimethoxy-2-isoquinolinyl)-ethyl]-phenyl}-9,10-dihydro-5-methoxy-9-oxo-4-acridine carboxamide [42]. Its molecular weight is 563.64 g/mol. Elacridar, an acridone carboxamide derivative, is a drug used in chemotherapy based on the tricyclic acridine structure [43]. Elacridar was developed and synthetically produced by the French company Glaxo as part of a research program to identify novel inhibitors of the mammalian P-gp protein in order to counter the MDR phenotype [43,44]. It is capable of competing with [3H]azidopine for the binding of P-gp, indicating that this transmembrane protein is the most probable target site [43]. The described mechanism of action concerns P-gp, but it is not specific, because elacridar is also a BCRP inhibitor.

Studies of elacridar are being conducted to re-sensitize cancer cells to treatment [45]. In two PAC-resistant gastric cancer cell lines that were characterized by established P-gp overexpression, the combination treatment composed of PAC and elacridar restored the antimitotic effect of PAC, sensitizing the cells to the cytotoxic drug [46]. A study was also conducted on human gastric carcinoma cells to overcome BCRP-dependent MDR. By encapsulating the chemotherapeutic drug SN-38 and elacridar within β-casein (β-CN) micelles, the ability to reverse MDR after simulated gastric digestion was demonstrated [47]. The synergistic effect of elacridar with imatinib re-sensitized imatinib-resistant cell lines expressing BCRP and P-gp in chronic myeloid leukemia (CML) [48]. In ovarian cancer and canine kidney cell culture with overexpression of BCRP, elacridar increased the efficacy of TOP treatment [49].

A study on rats has been conducted to analyze the effect of elacridar on the uptake of lapatinib by the brain and cerebrospinal fluid. It was shown that elacridar significantly increased lapatinib penetration into the cerebrospinal fluid and tissue of the brain [50]. The administration of elacridar and a tyrosine kinase inhibitor, vemurafenib, almost completely eliminated the role of BCRP and P-gp in vemurafenib penetration into the brain; such action can be used to treat brain metastases [51]. Other studies indicated that co-administration of TOP with elacridar leads to an increase in TOP bioavailability in a mouse model [52]. In tumors created by hepatocellular carcinoma (HCC) cell insertion in mice, treatment with elacridar combined with levatinib caused a significant antitumor effect compared to levatinib alone [53]. In another xenograft study, elacridar treatment reversed resistance to DOX in tumors formed from a DOX-resistant cell line P388/DOX [37]. By conducting studies in rats, optimal doses of elacridar application that inhibit BCRP and P-gp were determined. In rats, doses exceeding 8.9 mg/h/kg of elacridar effectively inhibited the blood–brain barrier efflux mediated by P-gp and BCRP, with no observed tolerability concerns [54]. In mice with Amyotrophic Lateral Sclerosis, the use of elacridar with riluzole affects the efficacy of riluzole in treatment, increasing the drug’s penetration into the central nervous system [55].

Clinical trials conducted on cancer patients have shown a role for elacridar in enhancing the bioavailability of TOP administered via the oral route [56,57]. In another patient study, elacridar considerably improved the bioavailability of orally administered PAC [37].

Two-dimensional culture models are commonly used to examine cell-specific mechanisms of MDR. Unfortunately, 2D culture models do not accurately replicate the tumor environment, where a dense cellular structure and overexpression of extracellular matrix (ECM) molecules predominate, resulting in limited drug diffusion [58].

The use of a 3D cell culture model greatly enhances the relevance of this study, due to its approximation to the conditions of an actual tumor. A 3D model of a spheroid formed under non-adhesive conditions was used in our study to simulate the tissue-specific mechanisms present in the tumor environment [59]. It reflects tissue-resistance mechanisms related to the histological structure of the tumor, like high-density cells and expression of ECM molecules [60,61]. Our previous experiments conducted with 3D models showed that cells cultured as spheroids are characterized by much greater resistance to chemotherapeutics, compared to the same cells cultured as monolayers [25,59,62]

As mentioned above, most ovarian cancer patients develop drug resistance during treatment [2]. Thus, a model of TOP resistance development in ovarian cancer was used to study the effect of elacridar on TOP resistance. We used A2780—a drug-sensitive ovarian cancer cell line—and two TOP-resistant cell lines: A2780TR1 and A2780TR2, characterized by very high levels of BCRP expression, which is the only drug transporter expressed in these cell lines [63]. To gain deeper insight into how elacridar overcomes resistance to cytotoxic therapy, we performed experiments on both TOP-sensitive and TOP-resistant cells under 2D and 3D culture conditions.

## 2. Results

### 2.1. The Drug Resistance Characterization of the A2780 Cell Line and TOP-Resistant Cell Lines

To better understand how elacridar affects drug resistance, we started our research by conducting a detailed analysis of drug resistance in the researched cell lines using the MTT cytotoxicity test. As we used TOP-resistant cultures, the first analyzed therapeutic was TOP. In the A2780TR1 cell line, a 20.50-fold increase in TOP resistance was observed, in comparison with the A2780 drug-sensitive cell line (IC50 = 200.68 ng/mL, compared to IC50 = 9.79 ng/mL). Similarly, a 13.48-fold higher resistance to TOP was noted in the A2780TR2 cell line (IC50 = 132.00 ng/mL, compared to IC50 9.79 ng/mL) (Table 1). The TOP-resistant cultures were also more resistant to another substrate of BCRP—MIT. We noted a 3.22-fold MIT resistance increase in the A2780TR1 cell line (IC50 = 9.60 ng/mL, compared to IC50 = 2.98 ng/mL) and a 3.24-fold resistance increase for the A2780TR2 cell line (IC50 = 9.65 ng/mL vs. IC50 = 2.98 ng/mL) compared to the TOP-sensitive culture (Table 1). Following CIS treatment of cells, the IC50 values showed no statistically significant variation. The IC50 values for CIS were 11,259 ng/mL for the A2780 cell line, 12,213 ng/mL for the A2780TR1 cell line, and 12,625 ng/mL for the A2780TR2 cell line (Table 1).

### 2.2. Characterization of BCRP Protein Expression in A2780 Cell Line and TOP-Resistant Cell Lines

**Figure 1:**
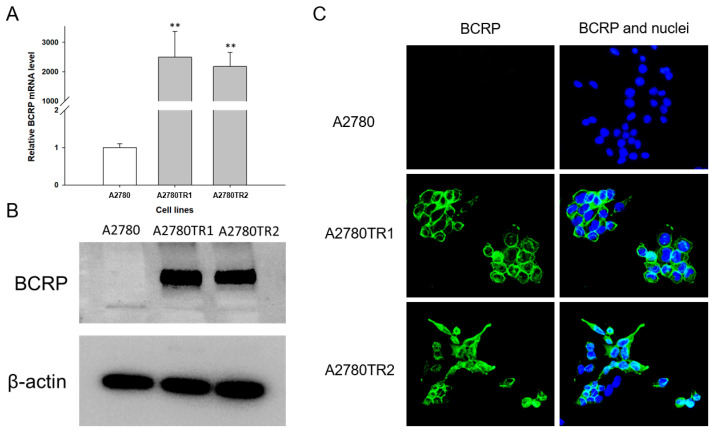
(**A**) Quantitative PCR analysis of BCRP mRNA expression in A2780 and TOP-resistant sublines. Relative expression levels in resistant cells (gray bars) are shown in comparison to the parental A2780 line (white bars), which was set as the reference value of 1. Statistical significance was considered at ** *p* < 0.01. (**B**) Western blot analysis of BCRP protein levels in A2780 and TOP-resistant cell lines. The cellular protein content was resolved on a 7% PAGE gel, transferred to a nitrocellulose membrane, and probed with a primary antibody against BCRP, followed by an HRP-conjugated secondary antibody. Detection of β-actin with a specific primary antibody served as a loading control. (**C**) Immunofluorescent staining of BCRP in A2780 and TOP-resistant cells. BCRP was visualized using an anti-BCRP primary antibody and an Alexa Fluor^®^488-labeled secondary antibody (green). Cell nuclei were counterstained with DAPI (blue). Scale bar: 50 μm.

As BCRP is the most important protein related to TOP resistance, we initially examined whether TOP-resistant ovarian cancer cells exhibited an altered expression of the BCRP gene/protein compared to the sensitive cell lines. The expression level of the *BCRP* gene examined by qPCR showed a statistically significant increase (*p* < 0.01) in expression in both TOP-resistant cell lines (Figure 1A). *BCRP* gene mRNA levels were over 2000-fold higher for the A2780TR1 and A2780TR2 cell lines than in sensitive cells. The presence of BCRP protein was investigated with the western blot method. Bands corresponding to this protein were detected only in TOP-resistant cell lines (Figure 1B). Immunocytochemical staining confirmed the presence of BCRP protein only in the A2780TR1 and A2780TR2 cell cultures (Figure 1C), localized mainly within the cell membrane.

### 2.3. BCRP Activity Analysis

**Figure 2:**
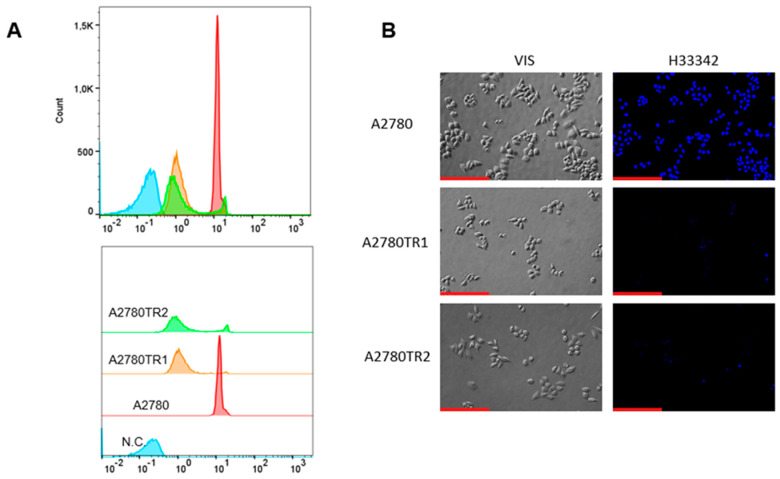
(**A**) Flow cytometry was used to assess intracellular accumulation of H33342 in both drug-sensitive and TOP-resistant cell lines. The fluorescence intensity histograms display data for the sensitive A2780 line (red) alongside the resistant sublines A2780TR1 (orange) and A2780TR2 (green). The negative control is shown in blue. (**B**) Fluorescence microscopy images illustrate H33342 accumulation (blue) within the drug-sensitive A2780 cells and the TOP-resistant A2780TR1 and A2780TR2 sublines. Scale bar: 200 μm.

**Figure 3:**
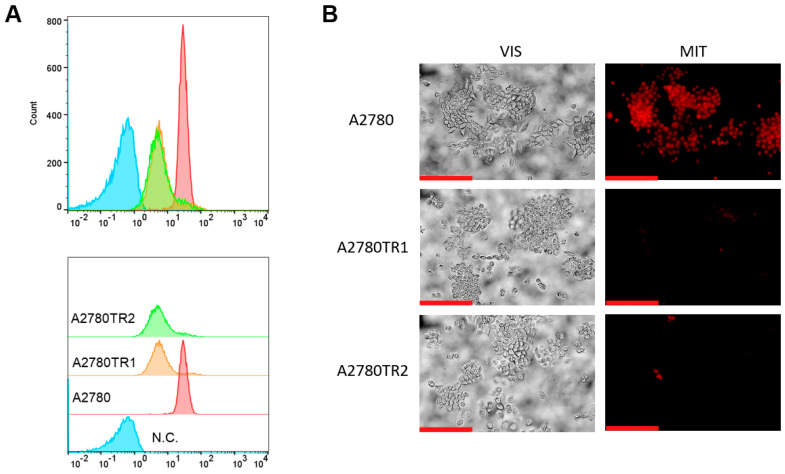
(**A**) Flow cytometry was used to assess intracellular accumulation of MIT in both drug-sensitive and TOP-resistant cell lines. The fluorescence intensity histograms display data for the sensitive A2780 line (red) alongside the resistant sublines A2780TR1 (orange) and A2780TR2 (green). The negative control is shown in blue (**B**) Fluorescence microscopy images illustrate MIT accumulation (blue) within the drug-sensitive A2780 cells and the TOP-resistant A2780TR1 and A2780TR2 sublines. Scale bar: 200 μm.

We investigated the BCRP protein activity by checking whether the ovarian cancer cells were able to accumulate the fluorescent BCRP substrates—H33342 and MIT. The accumulation of these compounds was investigated by flow cytometry analysis (Figure 2A and Figure 3A) and inverted fluorescent microscopy analysis (Figure 2B and Figure 3B). Using flow cytometry, in the case of both TOP-resistant sublines, we noted reduced H33342 and MIT fluorescence compared to A2780 drug-sensitive culture (Figure 2A and Figure 3A, respectively). The intravital imaging using the inverted fluorescence microscope confirmed flow cytometry results. In the A2780 drug-sensitive culture, we noted a clear accumulation of H33342 (Figure 2B) or MIT (Figure 3B). In contrast, both resistant cell lines did not exhibit H33342 (Figure 2B) or MIT (Figure 3B) accumulation.

### 2.4. Analysis of How Elacridar Affects the Resistance to Cytotoxic Drugs

**Figure 4:**
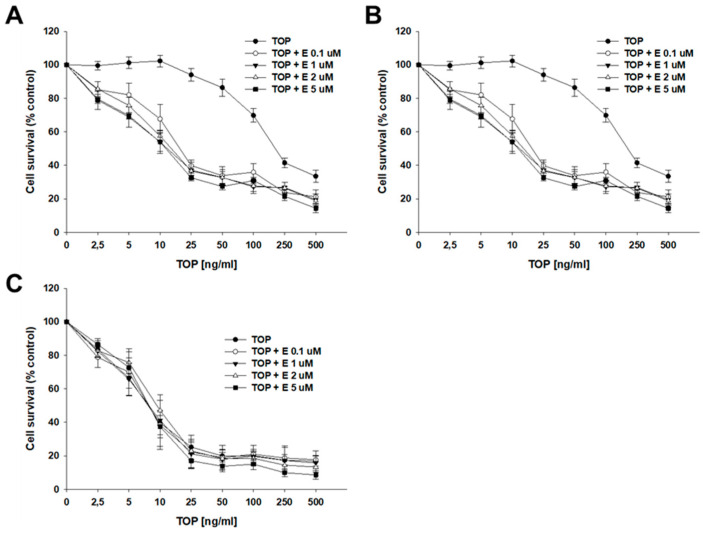
Elacridar (E) sensitizes TOP-resistant sublines to TOP in vitro. Cell lines resistant to TOP—A2780TR1 (**A**) and A2780TR2 (**B**) and the TOP-sensitive parental cell line A2780 (**C**) were cultured in 96-well plates. Afterward, they were treated for 72 h with TOP or TOP + E in concentrations of 0.1, 1, 2, and 5 μM. Following 72 h of treatment, the MTT assay was conducted to determine cell viability. Cell viability was calculated relative to the untreated control, expressed as a percentage. (mean ± SEM).

Next, we investigated whether elacridar, added in several different concentrations (0.1, 1, 2, and 5 µM), can inhibit BCRP protein activity and affect the cellular resistance to cytotoxic drugs. TOP was the first drug tested. In the drug-sensitive culture, we did not observe any significant change in IC50 value for TOP in the absence and presence of elacridar (Table 2, Figure 4C). However, in both TOP-resistant cell lines, the co-treatment with elacridar caused a reduction of the IC50 value in a manner dependent on elacridar concentration. The IC50 value for TOP in the A2780TR1 cell line was 204.68 ng/mL, and addition of elacridar decreased the IC50 value to 18.81 ng/mL (E—0.1 µM, 10.88 fold); 15.29 ng/mL (E—1 µM, 13.65 fold); 14.99 ng/mL (E—2 µM, 13.65 fold); and 12.05 ng/mL (E—5 µM, 16.98-fold), respectively. A similar effect was noted in the A2780TR2 cell line. The IC50 value for TOP in the A2780TR2 cell line was 132.00 ng/mL, and the addition of elacridar decreased the IC50 value to 19.11 ng/mL (E—0.1 µM, 6.91 fold); 11.11 ng/mL (E—1 µM, 11.88 fold); 10.02 ng/mL (E—2 µM, 13.17 fold); and 7.61 ng/mL (E—5 µM, 17.59-fold), respectively (Table 2). The responsive curve with and without the addition of elacridar is presented in Figure 4A (A2780TR1 subline) and Figure 4B (A2780TR2 subline).

**Figure 5:**
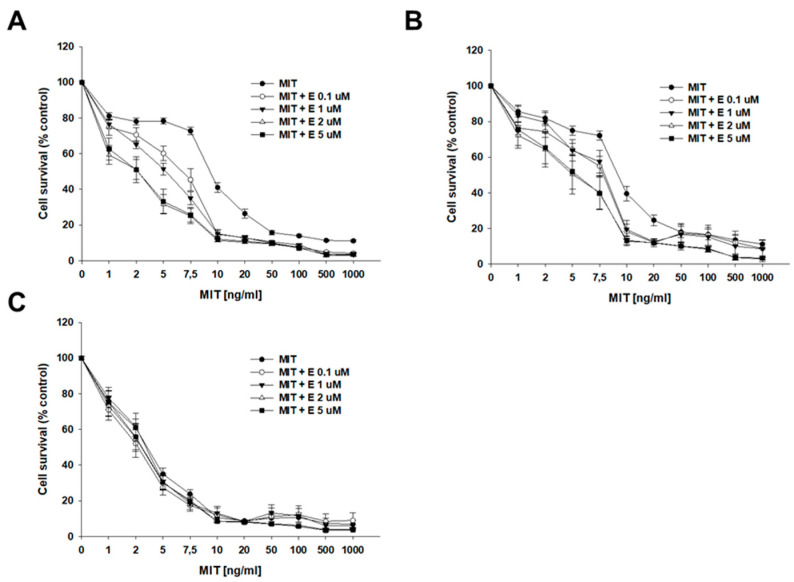
Elacridar (E) sensitizes TOP-resistant cell lines to MIT in vitro. Cell lines resistant to TOP—A2780TR1 (**A**) and A2780TR2 (**B**) and the TOP-sensitive parental cell line A2780 (**C**) were cultured in 96-well plates. Afterward, they were treated for 72 h with MIT or MIT + E in concentrations of 0.1, 1, 2, and 5 μM. Following 72 h of treatment, the MTT assay was conducted to determine cell viability. Cell viability was calculated relative to the untreated control, expressed as a percentage. (mean ± SEM).

MIT was the next drug that we studied (Figure 5, Table 3). In the drug-sensitive cell line, the IC50 value did not change significantly following the addition of elacridar. However, the IC50 values of both TOP-resistant lines decreased after co-treatment with MIT and elacridar. Namely, the IC50 value for the A2780TR1 cell line decreased 1.43-fold after the addition of 0.1 µM elacridar, 1.85-fold in the presence of 1 µM E, 3.93-fold in the presence of 2 µM E, and 3.55-fold in the presence of 5 µM E (IC50 = 9.60 ng/mL vs. 6.73 ng/mL vs. 5.25 ng/mL vs. 2.44 ng/mL vs. 2.71 ng/mL). For the A2780TR2 cell line, the effect of elacridar addition was less prominent than for the A2780TR1 cell line, but the changes in IC50 value were still statistically significant. The determined IC50 value changes for co-treatment with MIT and elacridar for the A2780TR2 cell line in comparison to untreated line are 1.32-fold lower for 0.1 µM E, 1.30-fold lower for 1 µM E, 1.90-fold lower for 2 µM E, and 1.93-fold lower for 5 µM elacridar (IC50 = 9.65 ng/mL vs. IC50 = 7.32 ng/mL vs. IC50 = 7.40 ng/mL vs. IC50 = 5.10 ng/mL vs. IC50 = 5.00 ng/mL) (Table 3). The responsive curves with or without elacridar are presented in Figure 5A (A2780TR1 cell line), Figure 5B (A2780TR2 cell line), and Figure 5C (A2780 cell line).

**Figure 6:**
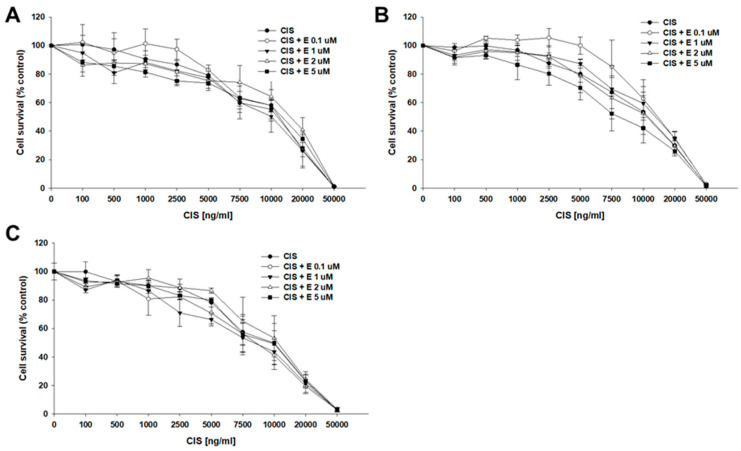
Effect of elacridar (E) on CIS-resistance in TOP-resistant cell lines in vitro. Cell lines resistant to TOP—A2780TR1 (**A**) and A2780TR2 (**B**) and the TOP-sensitive parental cell line A2780 (**C**) were grown in 96-well plates. Cells were treated for 72 h with either cisplatin (CIS) alone or in combination with elacridar (CIS + E) in concentrations of 0.1, 1, 2, and 5 μM. Following 72 h treatment, the cell viability was assessed using the MTT assay. Results were expressed as a percentage of the untreated control (mean ± SEM).

A similar experiment was performed to check if co-treatment of ovarian cancer cell lines with CIS and elacridar leads to changes in IC50 values (Table 4, Figure 6). In both the sensitive and TOP-resistant cell lines, no effect was observed in response to elacridar at any concentration on response to CIS treatment. For the A2780 cell line treated with CIS only, the IC50 value was 11,259 ng/mL and changed to 9228 ng/mL, 9470 ng/mL, 11,494 ng/mL, and 11,089 ng/mL, respectively, with elacridar concentrations of 0.1, 1, 2, and 5 µM. For the A2780TR1 cell line, the IC50 value for CIS alone was 12,213 ng/mL and it changed to 12,599 ng/mL (CIS + 0.1 µM E), 11,430 ng/mL (CIS + 1 µM E), 16,126 ng/mL (CIS + 2 µM E), and 13,686 ng/mL (CIS + 5 µM E). The established IC50 values for the A2780TR2 cell line were 12,625 ng/mL (CIS), 13,744 ng/mL (CIS + 0.1 µM E), 13,443 ng/mL (CIS + 1 µM E), 11,971 ng/mL (CIS + 2 µM E), and 9045 ng/mL (CIS + 5 µM E), respectively.

### 2.5. BCRP Protein Expression After Elacridar Treatment

**Figure 7:**
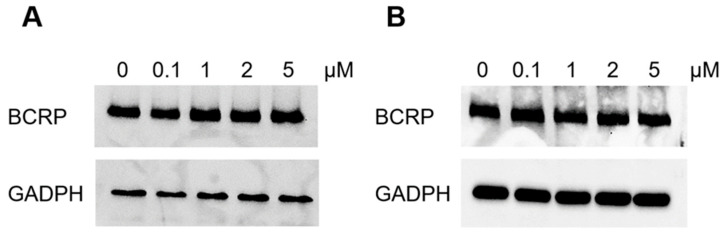
BCRP protein expression analysis in A2780TR1 (**A**) and A2780TR2 (**B**) after elacridar treatment at concentrations of 0, 0.1, 1, 2, and 5 μM. The cellular proteins were separated on a 7% PAGE gel and transferred to a nitrocellulose membrane, followed by immunoblotting with either primary Ab or HRP-conjugated secondary Ab. A primary anti-β Ab (A2780TR1) or GADPH (A2780TR2) was used as a loading control for the cell lysates.

As elacridar treatment caused increased sensitivity to BCRP substrates, we wanted to establish if elacridar treatment would change the expression of BCRP protein. Drug-resistant cell lines were cultured in the presence of elacridar at concentrations of 0.1 µM, 1 µM, 2 µM, and 5 µM, with cells without elacridar serving as a control. After 72 h, the expression of BCRP protein was investigated using the western blot technique. In both resistant cell lines, A2780TR1 (Figure 7A) and A2780TR2 (Figure 7B), we did not observe any effect of elacridar on BCRP protein expression.

### 2.6. BCRP Protein Activity After Elacridar Treatment in 2D Culture

**Figure 8:**
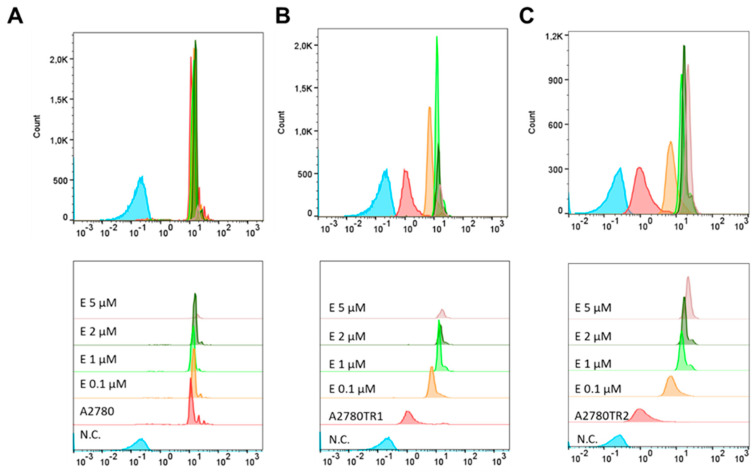
Effect of elacridar on intracellular H33342 accumulation by flow cytometry in drug-sensitive A2780 cells (**A**) and in TOP-resistant sublines A2780TR1 (**B**) and A2780TR2 (**C**). Fluorescence intensity profiles illustrate dye accumulation under different treatment conditions. Each panel compares untreated cells (red) with cells exposed to elacridar (E) at 0.1 μM (orange), 1 μM (green), 2 μM (dark green), and 5 μM (violet). A negative control sample lacking H33342 staining (N.C.) is shown in blue.

**Figure 9:**
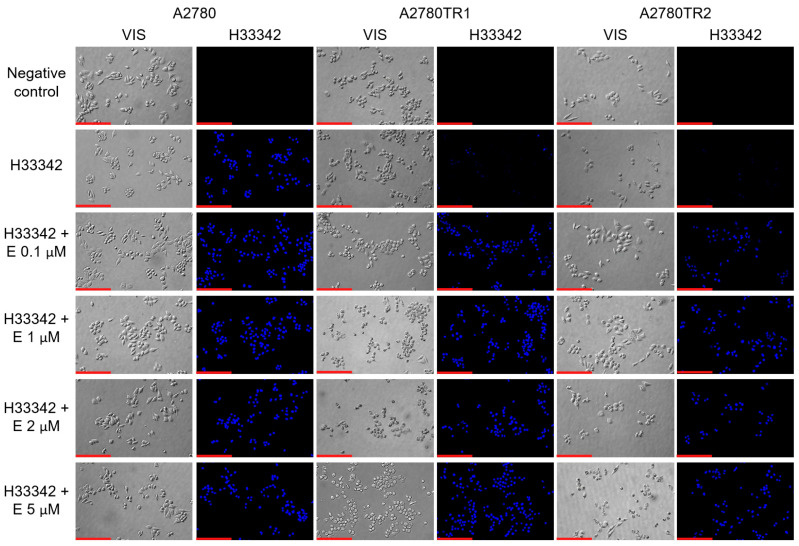
Fluorescent microscopy imaging of H33342 accumulation (blue) in drug-sensitive A2780 cell line and TOP-resistant sublines A2780TR1 and A2780TR2, with or without elacridar (E) treatment at concentration of 0.1, 1, 2, and 5 μM. Scale bar: 200 μm.

The effect of elacridar on BCRP protein activity was then tested using flow cytometry and inverted fluorescent microscopy methods. As previously, H33342 and MIT fluorescent dyes were used. TOP-sensitive and TOP-resistant cells were cultured with elacridar at concentrations of 0.1 µM, 1 µM, 2 µM, and 5 µM, with cells without elacridar treatment used as a control. Flow cytometry analysis with H33342 dye was performed (Figure 8). In the A2780 cells, we observed very similar fluorescence levels in with or without elacridar in any concentration used (Figure 8A). In contrast, in both TOP-resistant cell lines we observed an increase in fluorescence intensity in the cells treated with elacridar, and this effect seems to be dependent on elacridar concentration (Figure 8B,C). Next, we performed intravital imaging of H33342 dye accumulation using the inverted fluorescence microscope (Figure 9). In the A2780 cell line, we observed similar H33342 accumulation in cells in the absence and presence of elacridar, and increasing elacridar concentration did not change the fluorescence intensity of H33342 accumulated inside cancer cells. In both TOP-resistant cell lines cultured without elacridar, H33342 fluorescence was not observed. Treatment with 0.1 µM elacridar caused the accumulation of H33342 dye in the cells, with slightly lower fluorescence in A2780TR2 cells. Increasing elacridar concentration to 1 µM increased fluorescence intensity in the A2780TR2 cell line but not in the A2780TR1 cell line. The continued increase in elacridar concentration did not increase H33342 accumulation in A2780TR1 and A2780TR2 cell lines.

**Figure 10:**
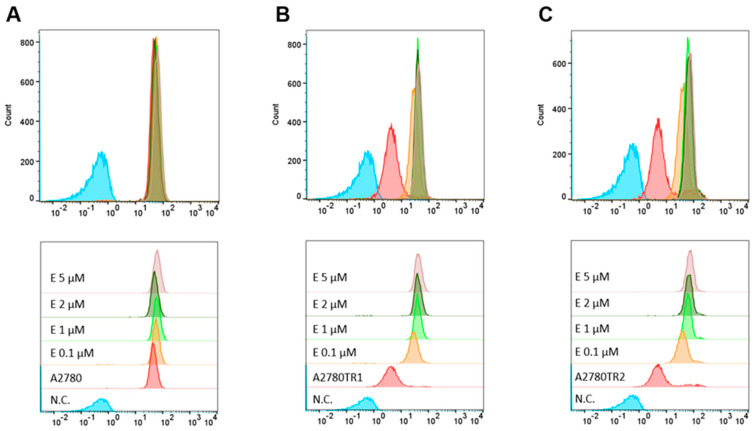
Effect of elacridar on intracellular MIT accumulation by flow cytometry in drug-sensitive A2780 cells (**A**) and in TOP-resistant sublines A2780TR1 (**B**) and A2780TR2 (**C**). Fluorescence intensity profiles illustrate dye accumulation under different treatment conditions. Each panel compares untreated cells (red) with cells exposed to elacridar (E) at 0.1 μM (orange), 1 μM (green), 2 μM (dark green), and 5 μM (violet). A negative control sample lacking H33342 staining (N.C.) is shown in blue.

To investigate the BCRP activity, we also used MIT, which has fluorescence activity. Flow cytometric analysis was performed to confirm the accumulation of MIT in the cells co-treated with elacridar. Treatment of the sensitive cell line with elacridar did not affect the accumulation of the MIT in the cells, even with the use of 5 µM elacridar (Figure 10A). Treatment of TOP-resistant cell lines with a concentration of 0.1 µM elacridar resulted in an increased fluorescence signal. Increasing the inhibitor concentration to 1 µM resulted in even greater accumulation of the fluorescent dye within the cells. Elacridar at concentrations of 2 µM and 5 µM did not increase MIT accumulation compared to the accumulation observed with an inhibitor at the concentration of 1 µM (Figure 10B,C).

**Figure 11:**
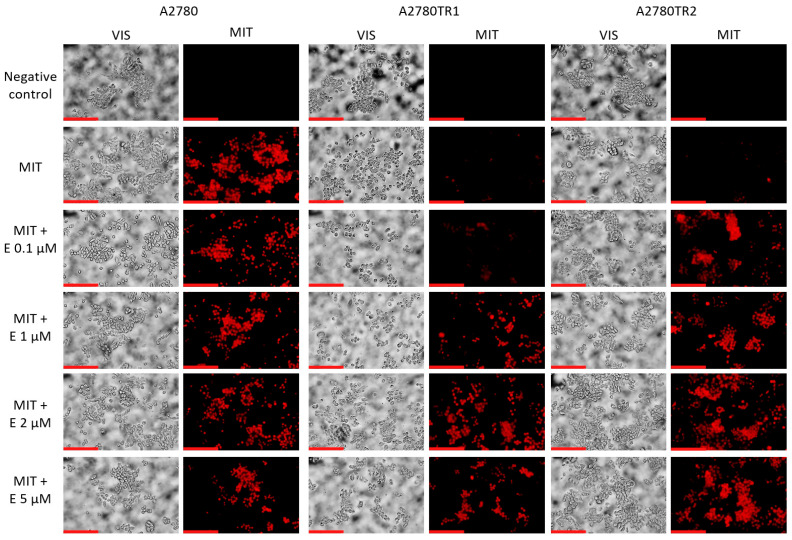
Fluorescent microscopy imaging of MIT accumulation (red) in drug-sensitive A2780 cell line and TOP-resistant sublines A2780TR1 and A2780TR2, with or without elacridar (E) treatment at concentrations of 0.1, 1, 2, and 5 μM. Scale bar: 200 μm.

Moreover, elacridar’s effect on MIT accumulation in ovarian cancer cells was also tested using fluorescence microscopy. Analysis showed that in the A2780 cell line, elacridar did not affect MIT accumulation at any tested concentration (Figure 11). In the drug-sensitive cell line, we noted similar accumulation of MIT in the absence and presence of elacridar. TOP-resistant cell lines do not show MIT accumulation when cultured without elacridar. Treatment with elacridar at a concentration of 0.1 μM caused a low fluorescence signal in some cells in the A2780TR1 cell line and a strong fluorescence signal in the A2780TR2 cell line. The addition of an inhibitor in a concentration of 1 µM caused a significant increase in MIT accumulation in the A2780TR1 cell line. Subsequent higher inhibitor concentrations (2 µM and 5 µM) did not further increase accumulation compared to the 1 µM concentration in both cell lines.

### 2.7. Analysis of Elacridar’s Effect in a 3D Model

**Figure 12:**
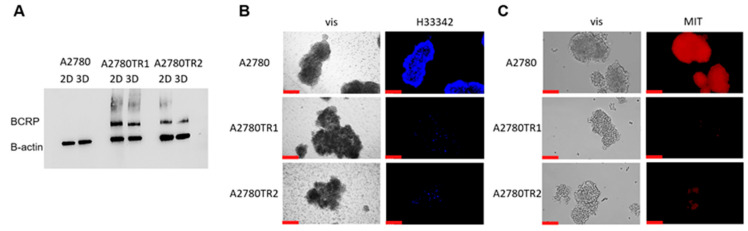
(**A**) Analysis of BCRP protein expression in the A2780 and TOP-resistant sublines grown in a monolayer (2D) or spheroids (3D). The cellular proteins were separated using a 7% PAGE gel and transferred to a nitrocellulose membrane, which was then immunoblotted with either primary Ab or HRP-conjugated secondary Ab. A primary anti-β Ab was used as a loading control for the cell lysates. (**B**) Fluorescent microscopy imaging of H33342 accumulation (blue) in 3D spheroids grown from the drug-sensitive A2780 cell line and TOP-resistant A2780TR1 and A2780TR2 sublines. (**C**) Fluorescent microscopy imaging of MIT accumulation (red) in 3D spheroids grown from the drug-sensitive A2780 cell line and TOP-resistant A2780TR1 and A2780TR2 sublines. Scale bar: 200 μm.

Cell culture in a monolayer does not reflect the conditions prevailing in the tumor, which is why experiments were also performed in the 3D cell culture model. First, we compared the BCRP protein level in cell lines growing in 2D and 3D cell cultures (Figure 12A). BCRP protein was not detected in the A2780 cell line under any of the culture conditions. In TOP-resistant cell lines, the presence of the BCRP protein band was observed and the expression level was independent of the model used.

The accumulation of the dyes H33342 and MIT in live spheroids was checked using inverted fluorescence microscopy. In spheroids generated from the A2780 cell line, we observed H33342 and MIT dyes’ accumulation, respectively (Figure 12B,C). In spheroids formed from both TOP-resistant cell lines, we did not observe a fluorescent signal in any of the dyes used (Figure 12B,C), suggesting high BCRP activity in spheroids formed from TOP-resistant cell lines.

**Figure 13:**
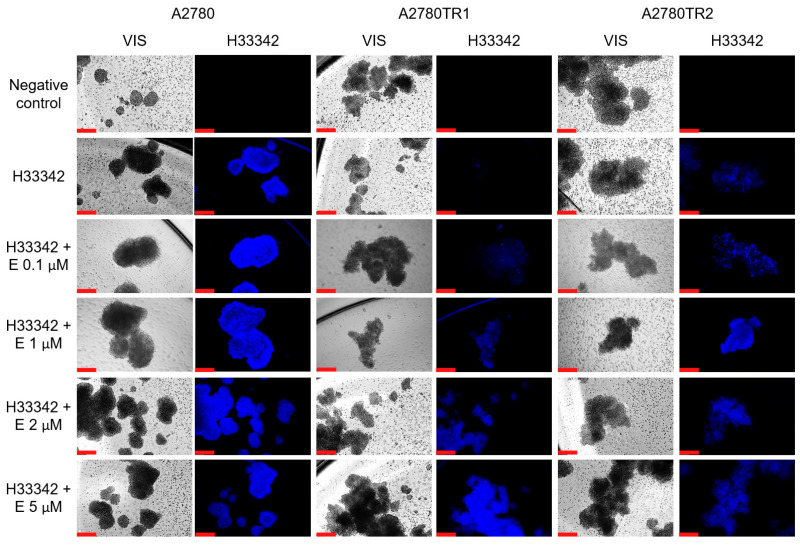
Fluorescent microscopy imaging of H33342 accumulation (blue) in spheroids grown from drug-sensitive A2780 cell line and TOP-resistant sublines A2780TR1 and A2780TR2, with or without elacridar (E) treatment in the concentration of 0.1 μM, 1 μM, 2 μM, and 5 μM. Scale bar: 200 μm.

Due to the lack of H33342 and MIT accumulation in spheroids from TOP-resistant cells, it was checked whether the treatment with elacridar would cause the inhibition of BCRP protein activity and dye accumulation in the 3D model. Spheroids from the A2780 cell line accumulate H33342 under standard culture conditions, and the addition of elacridar does not affect this accumulation. In spheroids generated from TOP-resistant cell lines, the accumulation of H33342 in the spheres increased in the presence of 0.1 µM elacridar, compared to the culture without inhibitor; however, greater accumulation was observed in the A2780TR2 cell line. Increasing the inhibitor concentration to 1 µM resulted in a further increase of H33342 accumulation, and the accumulation was greater in spheroids formed from the A2780TR2 cell line. Elacridar at 2 µM and 5 µM did not significantly increase H33342 accumulation in the spheres compared to the 1 µM concentration (Figure 13).

**Figure 14:**
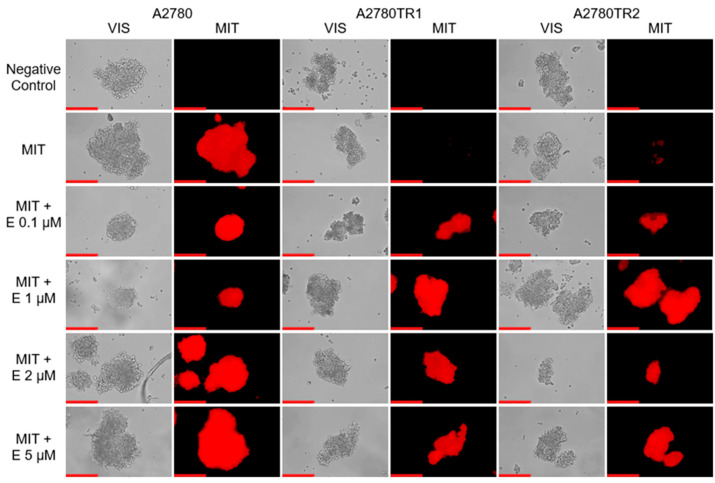
Fluorescent microscopy imaging of MIT accumulation (red) in spheroids grown from drug-sensitive A2780 cell line and TOP-resistant sublines A2780TR1 and A2780TR2, with or without elacridar (E) treatment in the concentration of 0.1 μM, 1 μM, 2 μM, and 5 μM. Scale bar: 200 μm.

The effect of elacridar on MIT accumulation in spheroids was also tested (Figure 14). Elacridar did not affect the accumulation of MIT in spheroids from the sensitive cell line. In contrast, we noted a clear effect of elacridar in a concentration of 0.1 µM on MIT accumulation in spheroids grown from TOP-resistant cell lines. Increasing the inhibitor concentration does not enhance the accumulation of the chemotherapeutic agent in spheroids.

**Figure 15:**
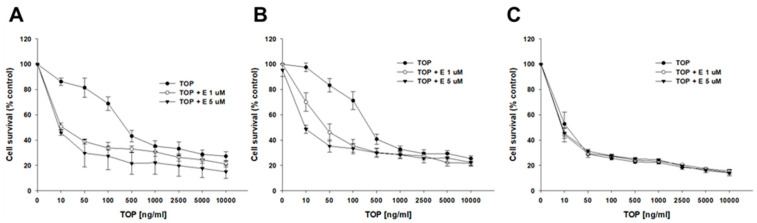
Effect of elacridar (E) on TOP resistance in drug-sensitive and TOP-resistant cell lines under 3D culture conditions. Spheroids were grown from 1 × 10^4^ cells of the TOP-resistant sublines: A2780TR1 (**A**) and A2780TR2 (**B**), as well as the TOP-sensitive parental A2780 cell line (**C**). Cultures were exposed to either TOP alone or in combination with elacridar (TOP + E) at concentrations of 1 μM or 5 μM for 72 h. Following treatment, cell viability was assessed using the MTT assay and reported as a percentage of the untreated control (mean ± SEM).

The effect of elacridar on TOP resistance was also examined in the 3D model. In this experiment, two inhibitor concentrations were selected—1 μM and 5 μM (Figure 15, Table 5). The treatment of spheroids generated from the A2780 cell line with elacridar did not cause significant changes in the determined IC50 values. The determined IC50 value for TOP treatment alone was 17.61 ng/mL. For cells treated with TOP and elacridar it was 11.71 ng/mL (1 µM elacridar) and 10.19 ng/mL (5 µM elacridar). In spheroids formed from TOP-resistant cells, the IC50 for TOP was significantly higher than for the A2780 cell line. It was 448.80 ng/mL (A2780TR1) and 404.38 ng/mL (A2780TR2), respectively. The treatment with elacridar significantly reduced the IC50 value in TOP-resistant sublines. For spheroids from the A2780TR1 subline, the IC50 was reduced to 15.10 ng/mL (29.66-fold) in the presence of 1 µM elacridar and to 9.27 ng/mL (48.31-fold) with 5 µM elacridar. The obtained IC50 value in spheroids grown from the A2780TR2 cell line was reduced to 62.63 ng/mL (6.46-fold) in the presence of 1 µM elacridar and to 16.02 ng/mL (25.24-fold) in the presence of 5 µM elacridar.

## 3. Discussion

Although cancer therapy with cytotoxic drugs is usually effective at first, cancers eventually acquire drug resistance during chemotherapy, rendering treatment ineffective [64]. The mechanisms leading to chemoresistance vary, although one of the most prominent is the overexpression of ABC proteins capable of active drug removal from the cancer cells. As a result, the required concentration of chemotherapeutic is not achieved [65]. BCRP, a known marker of cancer stem cells, is expressed in various cancers, including colorectal, ovarian, small cell lung cancer, and leukemias [66,67]. To combat the chemoresistance, known molecular inhibitors of ABC proteins are used in combination with traditional cytotoxic drug therapy, with the hope of re-sensitizing cancer to treatment [30,68]. One such inhibitor is elacridar, a potent P-gp and BCRP inhibitor [45].

To test the effect of elacridar on drug resistance, we chose the model of drug resistance development composed of the A2780 ovarian cancer cell line and two sublines resistant to TOP, A2780TR1 and A2780TR2 [22].

In the A2780TR1 and A2780TR2 cell lines, we noted a high TOP resistance level and a low increase in resistance to MIT. However, resistance to CIS was similar in the investigated cell lines. Different TOP and MIT resistance levels of can result from BCRP polymorphism. In the human population, there exist several polymorphic variants of BCRP that exhibit altered drug resistance, and in vitro experiments conducted in transfected HEK cells concluded that, for example, I206L mutation causes a 2-fold increase in TOP and MIT resistance, whereas D620N mutation decreases MIT resistance by 50% while not affecting TOP resistance [69]. Therefore, BCRP may affect certain drug resistances differently due to these mutations affecting protein function and affinity for certain drugs. Our TOP-resistant cell lines were selected using TOP, and for this reason, they preferentially developed TOP resistance and, to a lesser extent, MIT resistance.

As TOP resistance is associated with BCRP expression, we tested whether transcript and protein levels of BCRP were increased in these cell lines. We have confirmed that both resistant cell lines exhibit extremely heightened expression of BCRP, both at the transcript and protein levels, compared to the sensitive line, pointing out that BCRP is responsible for drug resistance in this model. Cell membrane localization of BCRP further confirmed its drug resistance function.

Next, we checked whether the BCRP expression level in our model correlates with the activity of this protein by treating the cells with known fluorescent substrates of BCRP—H33342 [70] and MIT [71]. Both H33342 and MIT can be successfully utilized to test BCRP activity or inhibition [21,66,72,73]. We then analyzed the fluorescence with flow cytometry and microscope observation of live cells—concluding that in both methods, the substrates were removed by BCRP in drug-resistant, BCRP-overexpressing cell lines and the substrates were retained by the cells in drug-sensitive culture.

In summary, these results confirm that BCRP overexpression and activity is an important factor of drug resistance in TOP-resistant cell lines. This is consistent with our [23] and others’ [21] previous results, where BCRP overexpression was observed in TOP-resistant cell lines. Thus, we selected this model to investigate the effect of elacridar on BCRP-dependent drug resistance.

The MTT assays performed on the model grown as a monolayer showed that elacridar, in all of the tested concentrations (0.1 μM, 1 μM, 2 μM, and 5 μM), causes resensitization of drug-resistant cells to TOP and MIT, known BCRP substrates [30], while not affecting the drug-sensitive cell line, suggesting that the effect of elacridar is BCRP-dependent. The higher the concentration, the better the results were generally observed, suggesting that the effect of elacridar is also concentration-dependent. On the other hand, elacridar did not cause re-sensitization of drug-resistant cell lines to CIS, as CIS is not a BCRP substrate [74].

Elacridar’s effect on sensitivity to cytotoxic drugs has also been investigated by others [45]. Cell viability assays conducted in other works determined elacridar’s ability to decrease cell survival when combined with imatinib [48], a cancer medication that is a known BCRP substrate [75]. The experiments were carried out on chronic myeloid leukemia (CML) cell lines, both sensitive (K562 and LAMA-84) and the derived drug-resistant, P-gp- and BCRP-over expressing cell lines (K562-RC and K562-RD), where it was determined that 0.25 µM elacridar combined with various imatinib concentrations decreased cell viability in a dose- and time-dependent manner [48]. Similarly, another assay method revealed that cotreatment of the cells with TOP and elacridar in a concentration of 5 µM significantly decreased the IC_50_ of treated cells—by 32-fold in the BCRP-expressing, drug-resistant human ovarian adenocarcinoma IGROV1-derived T8 cell line and by 5-fold in the canine kidney MDCKII-BCRP1 cell line—compared to cells treated with TOP alone [49]. Other assays conducted on the drug-resistant, BCRP-overexpressing ovarian cancer cell lines T8 and MX3 confirmed that elacridar added in a concentration of 2 µM allowed to re-sensitize both the cell lines to TOP (IC50 decreased 27-fold for T8 and 20-fold for MX3) [76]. Elacridar in a concentration of 0.5 µM was also capable of decreasing the drug resistance of the BCRP-overexpressing KYN-2 (human pleomorphic HCC) cell line, increasing sensitivity to CPT-11 and SN-38 drugs, as well as in Lovo (human colorectal cancer) and H23 (human adenocarcinoma) cell lines that express high amounts of BCRP mRNA [77]. Elacridar was proven to greatly increase sensitivity to MIT in BRCP-overexpressing human colon carcinoma cell lines S1-M1-80, where it was used in concentrations of 0.5 µM (causing a 141-fold sensitivity increase) and 1 µM (causing a 1850-fold sensitivity increase) [78]. In experiments conducted on BCRP-overexpressing human gastric carcinoma cell line EPG85-257RNOV, a combination of SN-38 and 1 µM elacridar caused over 9-fold increase in cell sensitivity, compared to the cell line treated with SN-38 alone [47].

The functional experiments performed with elacridar and fluorescent substrates of BCRP H33342 and MIT confirmed that elacridar inhibits BCRP activity in TOP-resistant cell lines in a concentration-dependent manner, which corresponds with cytotoxicity test results. Although elacridar caused BCRP activity inhibition, it did not alter BCRP expression, which was proven by western blot analysis.

Elacridar’s ability to inhibit BCRP function was tested by others in 2D conditions with Hoechst as a fluorescent substrate in HEK-R2 cells transfected to overexpress BCRP, where the inhibition was stronger with higher concentrations used (the tested concentrations were 10 nM, 50 nM, and 1 μM) [79]. In other work, flow cytometry analysis confirmed that elacridar in the concentration of 0.5 µM increased cell accumulation of chemotherapeutic drug CPT-11 in BCRP-expressing human pleomorphic HCC cell line KYN-2 [77]. The MIT efflux assay conducted on MCF-7/Top breast cancer, TOP-resistant cell lines confirmed that elacridar inhibits BCRP function in a concentration-dependent manner [80].

In summary, we and others observed a concentration-dependent effect of elacridar in a range of micromolar concentrations on BCRP activity and resistance to drugs that are BCRP substrates. However, the increase in drug sensitivity was dependent on the cell line and the type of investigated drug.

The obtained results indicate that elacridar suppresses BCRP activity and enhances the sensitivity of drug-resistant cancer cells to cytotoxic agents that are BCRP substrates. This suggests that elacridar could be a promising candidate for improving the efficacy of chemotherapy. However, the experiments mentioned above were performed using 2D cell cultures, which are optimal for investigating cellular mechanisms of drug resistance but do not fully replicate the complex conditions of tumors, such as high cell density and the presence of an extracellular matrix [81]. To account for these conditions, we conducted similar experiments using cell cultures grown as 3D spheroids. This model, which we had previously characterized and described in detail, provides a more physiologically relevant environment for studying drug resistance [62,82].

The initial evaluation of BCRP protein expression confirmed that drug-resistant cell cultures exhibit elevated BCRP levels under both 2D and 3D growth conditions. Additionally, no significant differences in BCRP quantity were observed between samples derived from 2D and 3D cell lysates. These findings align with our previous study, which reported similar BCRP gene expression levels in both 2D and 3D cell culture models and expression of BCRP protein in 3D spheroids detected using immunohistochemistry [82]. Therefore, BCRP overexpression appears to be a constant feature of drug-resistant cells, regardless of their growth conditions.

To investigate BCRP activity in live spheroids, we utilized inverted fluorescence microscopy. Analysis of spheroids treated with Hoechst 33342 and MIT revealed that both substrates were actively removed from spheroids derived from TOP-resistant cell lines, indicating strong BCRP activity in these aggregates. In contrast, spheroids formed from the A2780 cell line exhibited high fluorescence, suggesting that the substrates were able to penetrate into the spheroid structure.

Next, we wanted to check if elacridar can inhibit BCRP activity in 3D spheroids. In the case of H33342 dye, the effect of elacridar seems to be concentration-dependent, while in the case of MIT, similar fluorescence in all elacridar concentrations was observed. These differences can result from different abilities of both dyes to penetrate through the spheroid structure and their different affinity to BCRP. In summary, inhibition of BCRP activity with elacridar resulted in the accumulation of BCRP substrates in the spheroids. In our previous study, we also observed that elacridar very effectively inhibits P-gp activity in 3D spheroids, resulting in a high level of Calcein-AM accumulation [83]. In other works, the 3D spheroid model of HEK cells transfected to overexpress BCRP was used to confirm elacridar’s ability to inhibit BCRP function, with Hoechst 33342 being the fluorescent substrate; moreover, this experiment concluded that out of all the tested BCRP inhibitors (ko143, iressa, and elacridar), the latter was the only one to induce a long-lasting (over 5 h) inhibitory effect following washing and transferring spheres to a new hydrogel without the drug [79].

All these results indicate that elacridar is able to penetrate into the spheroid structure and efficiently inhibit BCRP activity. This makes an elacridar a potent anti-cancer drug, increasing the effectiveness of chemotherapy. To verify this hypothesis, we investigated the effect of elacridar on TOP cytotoxicity in 3D spheroids. A significant effect of elacridar was observed in spheroids formed from the A2780 cell line. Both TOP-resistant cell lines showed a similar level of TOP resistance in 3D conditions. Inhibition of BCRP activity with 1 µM elacridar resulted in a nearly 30-fold decrease in IC50 value for TOP in spheroids from the A2780TR1 cell line to a level similar to that of the A2780 TOP-sensitive cell line. However, only a 6-fold decrease in IC50 value in spheroids from the A2780TR2 cell line was observed at this inhibitor concentration. An increase in elacridar concentration to 5 µM resulted in a further decrease of the IC50 value in both cell lines to a level similar to that of the A2780 TOP-sensitive cell line. As both TOP-resistant spheroids have a similar level of BCRP expression, the differences may result from other factors like spheroid density or the presence of other drug resistance mechanisms in A2780TR2 cell lines.

In summary, the 3D study showed that elacridar effectively inhibits BCRP activity in 3D spheroids, resulting in decreased resistance to TOP to a level similar to that in a drug-resistant cell line. This makes an elacridar a potential drug that can increase the effectiveness of chemotherapy based on TOP and other BCRP substrates. This also indicates that results obtained in 2D monolayers should be further verified in 3D cell culture models that are much more similar to real tumor conditions.

Other studies also appear to confirm the utility of elacridar as a potential anti-cancer drug. In xenograft experiments, where tumors were generated using HCC cells, combining elacridar with lenvatinib led to a much stronger antitumor response compared to lenvatinib alone [53]. Another study showed that elacridar was able to overcome DOX resistance in tumors derived from the resistant P388/DOX cell line [37]. In animal trials conducted on mice, elacridar increased the bioavailability of TOP compared to the control without elacridar [52]. Other experiments with elacridar conducted on WT mice revealed that it can successfully inhibit both P-gp and BCRP activity, leading to the increase of dasatinib accumulation in the brain [84]. In ALS mice, elacridar improved riluzole penetration into the CNS, enhancing its therapeutic effect [55]. In rats, elacridar greatly enhanced the penetration of lapatinib into both the cerebrospinal fluid and brain tissue [50]. Other rat studies helped determine optimal elacridar doses for BCRP and P-gp inhibition at the blood–brain barrier without tolerability concerns [54]. Co-administration of elacridar and the tyrosine kinase inhibitor vemurafenib almost completely eliminated the role of BCRP and P-gp in vemurafenib brain penetration, which may prove to be useful in treating brain metastases [51]. Elacridar’s ability to inhibit BCRP function was also tested in Phase I clinical trials, where it was determined that therapy consisting of 1mg oral TOP with 1000 mg elacridar increased systemic exposure to TOP, with apparent bioavailability rising significantly from 40% to over 97% [57]. Another Phase I study concluded that 2.0 mg oral TOP administered with 100 mg elacridar results in the complete oral bioavailability of TOP [56]. Clinical trials in cancer patients also demonstrated that elacridar enhances the bioavailability of orally administered PAC [37].

In conclusion, we investigated the potential of elacridar—a third-generation, dual P-gp and BCRP inhibitor—in cytotoxic drug-resistant ovarian cancer cell lines. Our 2D experiments demonstrated that elacridar strongly suppresses BCRP activity in a concentration-dependent manner and enhances drug response to TOP and MIT, both known BCRP substrates. In contrast, no change in sensitivity was observed for CIS, which is not a BCRP substrate. This aligns with previous findings [77,80]. The experiments conducted in 3D spheroids also confirmed effective BCRP inhibition and sensitization of spheroids to TOP following elacridar treatment. Studies conducted on xenografts, animals, and in clinical trials also appear to confirm the potential of elacridar as an anticancer drug, capable of increasing the bioavailability of drugs and enhancing the response of tumors to various cytotoxic drugs [52,53].

However, continued experiments are necessary to fully determine the usefulness of this inhibitor in ovarian cancer treatment.

## 4. Materials and Methods

### 4.1. Reagents and Antibodies

The cell culture reagents of fetal bovine serum (FBS), MEM medium, penicillin, streptomycin, L-glutamine, amphotericin B (25 μg/mL), and trypsin-EDTA solution were obtained from Sigma (St. Louis, MO, USA). DPBS was purchased from Corning (Corning, NY, USA). Cisplatin (CIS), TOP, MIT, and elacridar were obtained from Selleckchem (Houston, TX, USA). Cell proliferation Kit I (MTT)—Thiazolyl Blue Tetrazolium Bromide and bisBenzimide H33342 trihydrochloride (H33342) were purchased from Sigma (St. Louis, MO, USA).

Rabbit anti-BCRP/ABCG2 antibody (D5V2K) was obtained from Cell Signaling (Danvers, MA, USA). The secondary antibodies of HRP-conjugated Goat Anti-Rabbit IgG (SA00001-2), goat anti-mice HRP-conjugated (SA00001-1-A), anti-β-actin (66009-1-Ig), and anti-GAPDH (60004-1-Ig) were purchased from Proteintech (Rosemont, IL, USA), and Alexa Fluor^®^ 488 AffiniPure™ Donkey Anti-Rabbit IgG (H+L) (711545-152) was purchased from Jackson ImmunoResearch Laboratories, (Ely, Cambridgeshire, UK). All western blot reagents (including gels, protein markers, and membranes) were purchased from Bio-Rad (Bio-Rad Laboratories, Hemel Hempstead, UK). qPCR reagents, like the M-MLV reverse transcriptase kit (28025013) and RnaseOUT (10777019), were acquired from Invitrogen by Thermo Fisher (Waltham, MA, USA).

### 4.2. Cell Lines and Cell Culture

The human ovarian carcinoma cell line A2780 was purchased from American Type Culture Collection (ATCC, Manassas, VA, USA). The A2780 sublines, resistant to TOP (A2780TR1 and A2780TR2), were obtained by the stepwise selection of A2780 cells exposed to TOP in increasing concentrations. The final concentration used for selecting the resistant cells was 24 ng/mL of TOP.

The cell lines were cultured in MEM medium with 10% (*v*/*v*) fetal bovine serum content and 2 mM L-glutamine, as well as streptomycin (100 U/mL), penicillin (100 U/mL), and amphotericin B (25 µg/mL), under standard culture conditions (5% CO_2_ atmosphere at 37 °C). TOP (final concentration—24 ng/mL) was added to the cultures of resistant cell lines.

### 4.3. Isolation of the RNA, cDNA Synthesis, and qPCR

The RNA was isolated using Gene Matrix Universal RNA Purification Kit (EURx, Ltd., Gdańsk, Poland) according to the isolation protocol. The concentration of the obtained RNA was determined by measuring the absorbance (260 nm and 280 nm), and then cDNA was synthesized using the CFX Opus 96 Real-Time PCR System (Bio-Rad Laboratories, Hemel Hempstead, UK). Each sample consisted of 1.5 μg RNA, 1 μL oligodT18A (IBB PAN, Warsaw, Poland), and 1 μL dNTP Mix (ThermoFisher, Waltham, MA, USA, R0192). The samples were denatured at 65 °C for 5 min. Next, 4 μL 5X First Strand Buffer, 2 μL DTT, 0.5 μL RnaseOUT, and 0.5 μL M-mLV RT were added to each sample and the reaction was performed for 60 min at 37 °C and 15 min at 75 °C.

Real-time PCR reaction mixture for one sample was prepared by mixing 12.5 μL of TakyonTM ROX SYBR^®^ MasterMix blue dTTP from Eurogentec (Searing, Belgium), 1 μL of each sequence-specific primer (7.5 µM) from Oligo.pl (Warsaw, Poland) (Table 6), 9.5 μL of nuclease-free water, and 1 μL of cDNA solution. Nuclease-free water was used instead of cDNA in the negative control and GPDH was used as a reference gene. Real-time PCR was performed using a CFX Opus 96 Real-Time PCR System (Bio-Rad Laboratories, Hemel Hempstead, UK) under the following conditions: 95 °C for 15 min (initial denaturation), 45 cycles, 95 °C for 15 s (denaturation), 60 °C for 30 s (primer annealing), 72 °C for 30 s (extension); and 72 °C for 30 s (final extension).

The analysis of the obtained results was performed in the CFX Maestro (Bio-Rad v2.3 (5.3.022.1030) software.

Next, we performed the analysis of gene expression. The relative quantification (RQ) method was used and the differences in gene expression were evaluated relative to a calibrator (RQ of the calibrator = 1). The drug-sensitive cell line A2780 was used as a calibrator in this study. We then calculated the RQ values using the standard formula RQ = [sample (drug-resistant line) calibrator (drug-sensitive line)]. We generated the graphical representations of the results with SigmaPlot software 15.0 (Systat Software, Inc., San Jose, CA, USA).

### 4.4. Treatment of Cells with Elacridar

The cell lines were seeded into the 6-well plates at 2 × 10^5^ cells per well in a volume of 2000 µL culture medium. After 48 h, the cells were treated with elacridar in concentrations of 0.1, 1, 2, and 5 µM. The cells were cultured with the inhibitor for 72 h. Next, protein isolation and western blot analysis were performed as described below.

### 4.5. Protein Isolation and Western Blot Analysis

Protein isolation was performed after washing cells with Phosphate-Buffered Saline (PBS) with Ca/Mg ions three times. The spheroids were dissociated by vigorous pipetting, then washed three times with calcium/magnesium-free PBS, followed by centrifugation at 200× *g* for 10 min. Cells (1 × 10^6^ cells/300 µL lysis buffer) were lysed using RIPA buffer containing a protease inhibitor cocktail (Roche Diagnostics GmbH, Mannheim, Germany). Incubation was carried out for 80 min at 4 °C, and then the cells were centrifuged at 13.4 × 10^3^ rpm for 30 min at 4 °C. Protein concentrations were determined using the Bradford protein assay system (Bio-Rad Laboratories, Hemel Hempstead, UK). Protein samples for separation included 7 µg protein, 4× loading buffer (Bio-Rad Laboratories, Hemel Hempstead, UK), and 10% β-mercaptoethanol. Protein separation was performed on a 4–20% mini-PROTEAN^®^ TGX™ precast gel using the SDS-PAGE technique. Next, the proteins were transferred to a nitrocellulose membrane using the Trans-Blot^®^™ transfer system (Bio-Rad Laboratories, Hemel Hempstead, UK), blocked with 5% solution of non-fat dry milk in TBS/Tween (0.1 M Tris-HCl, 0.15 M NaCl, 0.1% Tween 20) for 1 h and incubated overnight in primary antibodies against BCRP (1:2000), followed by a 3 h incubation with goat HPR-labeled secondary antibody (1:10,000). Afterwards, the membranes were washed with TBS/Tween (0.1 M Tris-HCl, 0.15 M NaCl, 0.1% Tween 20) and incubated with antibodies, either against β-actin (1:7500) or against GADPH (1:40,000), followed by a 3 h incubation with goat HPR-labeled secondary antibody (1:10,000). The signals were developed using chemiluminescence detection system (ECL, Femto Super Signal Reagent) and ChemiDoc™ (Bio-Rad Laboratories, Hemel Hempstead, UK). The β-actin or GADPH was used as a reference protein.

### 4.6. Immunofluorescence

The cells were cultured in a 24-well plate on coverslips until 70% confluence was achieved. After removing the medium and washing the cells with PBS, they were fixed and permeabilized with ice-cold acetone/methanol (1:1) solution for 15 min at room temperature (RT). Cells were then washed with PBS, blocked with 3% BSA solution for 30 min at RT, and incubated with the primary antibody, anti-BCRP (1:300), for 2 h at RT. After washing the cells three times with PBS, they were incubated with a secondary antibody (Alexa Fluor^®^488-conjugated Goat anti-rabbit) for 1 h at RT. Slides with cells were washed three times with PBS, then once with distilled water, and covered with mounting medium with DAPI (Sigma, F6057) to visualize cell nuclei. BCRP expression was analyzed using a fluorescence microscope (Zeiss Axio-Imager.Z1, Oberkochen, Germany) and Zen Blue v3.3 software. Objective 40× was used.

### 4.7. Live-Cell Immunofluorescence (H33342 Accumulation)

Cell lines were seeded in 24-well plates at 1 × 10^4^ cells per well. After 72 h, the cells were incubated in H33342 solutions (0.5 μg/mL) for 1 h. One hour later, the cell lines were washed three times with a cold solution of 50 μM Verapamil in PBS. The cells were observed under an inverted fluorescence microscope, a Leica DMi8, with a magnification of 20× for DAPI and VIS channels, and pictures were taken for each condition.

H33342 accumulation was also examined following the treatment of cells with elacridar. The cells were prepared in the same manner as described above. They were then treated with the appropriate concentration of elacridar for 1 h. Then, the medium was replaced with one containing the proper concentration of elacridar and H33342. After an hour of incubation, the cells were washed three times with a solution of 50 μM Verapamil in PBS and observed under an inverted fluorescence microscope, a Leica DMi8, at a magnification of 20× for DAPI and VIS channels, and pictures were taken.

Life-cells immunofluorescence (MIT accumulation)

Cell lines seeded at 1 × 10^4^ cells per well were cultured in a 24-well plate. After 72 h, the cells were incubated with the MIT solution (5 μg/mL) for 1 h. After an hour, the cell lines were washed three times with a cold solution of 50 μM Verapamil in PBS. The cells were observed under the inverted fluorescence microscope Leica DMi8, supported by Leica Application Suite X (Leica Microsystems GmbH, Wetzlar, Germany), with a magnification of 20× for Fluo-Red Y5 and VIS channels.

MIT accumulation was also examined following the treatment of cells with elacridar. The cells were prepared in the same manner as described above and treated with the appropriate concentration of elacridar for 1 h. Then, the medium was replaced with one containing the proper concentration of elacridar and MIT. After an hour of incubation, the cells were washed three times with a solution of 50 μM Verapamil in PBS and observed under the fluorescence microscope Leica DMi8, supported by Leica Application Suite X (Leica Microsystems GmbH, Wetzlar, Germany), with a magnification of 20× for Fluo-Red Y5 and VIS channels.

### 4.8. Flow Cytometry Analysis

To check the activity of the BCRP protein, the H33342 accumulation assay was performed. The cell suspensions (1 × 10^6^ cells/mL in cell culture medium) were treated with elacridar (0.1, 1, 2, 5 µM) or with medium alone, and then incubated for 1 h at 37 °C with mixing (800 rpm). Next, H33342 in the final concentration of 0.5 μg/mL was added, and the cells were incubated under the same conditions for 1 h. As a negative control, we used the cells untreated with elacridar and H33342. After one hour of incubation, the cells were placed on ice, centrifuged at 200× *g* for 5 min at 4 °C, and washed twice in ice-cold PBS with 50 μM verapamil. Afterwards, the data was collected using a MACSQuant 10 cytometer and then exported to the FACS-FlowJo 10.9 software for analysis. For each analysis, 10,000 events were recorded. The fluorescent emission was collected at 405 nm.

To check the activity of the BCRP protein, the accumulation of the cytotoxic agent MIT was also performed. The cell suspensions (1 × 10^6^ cells/mL in cell culture medium) were treated with elacridar (0.1, 1, 2, or 5 µM) or with medium alone, and then incubated for 1 h at 37 °C with mixing (800 rpm). Next, MIT in the final concentration of 0.2 μg/mL was added and the cells were incubated under the same conditions for 1 h. As a negative control, we used the cells untreated with elacridar and MIT. After one hour of incubation, the cells were placed on ice, centrifuged at 200× *g* for 5 min at 4 °C, and washed twice in ice-cold PBS with 50 μM verapamil. Afterwards, the data was collected using a MACSQuant 10 cytometer and then exported to the FACS-FlowJo 10.9 software for analysis. Each time, we recorded 10,000 events. We collected the fluorescent emission at 640 nm.

### 4.9. Analysis of MIT and H33342 Accumulation in Spheroids

The cells were cultured in the non-adherent 96-well plates (BRAND plates inter Grade, F-bottom, 781902, Merck, Darmstadt, Germany))—at 1 × 10^4^ cells per well. The spheroids were cultured for 5 days. Then, they were treated with the proper dosage of elacridar for 1 h. Next, the cells were treated with the same concentration of elacridar again, with the addition of 0.5 µM H33342 for 1 h. Afterwards, the spheroids were washed with a cold solution of 50 μM Verapamil in PBS. Spheroids were observed under a fluorescence microscope (Leica DMI8) at 10× magnification for the DAPI and VIS channels.

A similar experiment was performed for MIT- and elacridar-treated spheroids. The cells were cultured for 2 days to obtain stable spheroids. After this time, the cells were treated with a mixture of MIT (2 µg/mL) and elacridar at appropriate concentrations. After 72 h, the spheroids were observed under a fluorescence microscope (Leica DMI8) with 20× magnification, for the Fluo-Red Y5 and VIS channels.

### 4.10. 2D MTT Assay

TOP-sensitive and TOP-resistant cell lines were cultured in the 96-well plates at 3 × 10^3^ cells per well in a volume of 200 µL culture medium. After 48 h, the cells were treated with a mixture of elacridar (0.1, 1, 2, and 5 µM) and appropriate concentrations of TOP, MIT, or CIS. The cells were cultured with the inhibitor and cytotoxic agents for 72 h. Then, the medium was removed from the cells and the mixture of 100 µg MTT with 170 µL medium was added to each well (total MTT concentration was 0.59 µg/mL). Following a 1 h incubation, the medium was replaced with 200 µL of DMSO, in which formazan crystals dissolved. Absorbance was recorded at 570 nm and 720 nm using a Synergy LX Multi-Mode Reader (BioTek Instruments, Inc.). Each experiment was performed in duplicate and replicated at least four times. The resulting data were used to calculate the IC50 values.

### 4.11. 3D MTT Assay

Cell cultures were seeded into nonadherent 96-well plates at 1 × 10^4^ cells per well. After 48 h, 100 µL of the medium was replaced with a mixture of elacridar (1 or 5 µM) and appropriate concentrations of TOP. After 72 h of incubating the spheroids with the drugs and inhibitor, cell viability was assessed using the MTT assay with the Cell Proliferation Kit I (Roche, 11465007001). From each well, 100 µL of medium was removed, and 10 µL of MTT reagent was added (total MTT concentration was 0.45 µg/mL). Following a 4 h incubation, 100 µL of solubilization buffer was introduced, and the plates were incubated overnight at 37 °C. Absorbance was measured at 570 nm and 720 nm using a Synergy LX Multi-Mode Reader (BioTek Instruments, Inc., Winooski, VT, USA). Each experiment was performed in duplicate and replicated at least four times. IC50 values were calculated based on the obtained absorbance data.

### 4.12. Statistical Analysis

Data from the MTT assay and qPCR were analyzed using Student’s *t*-test and are presented as the mean ± standard error of the mean (SEM). A *p*-value of less than 0.05 was considered statistically significant. Each experiment was repeated at least four times.

## Figures and Tables

**Table 1:** Overview of cell line responses to TOP, MIT, and CIS treatments under 2D culture conditions determined by MTT test. The mean IC50 values for each drug are provided for all tested cell lines, with the range presented in brackets. For reference, the drug resistance of the parental A2780 line is normalized to 1. Underlined figures represent fold changes in resistance relative to A2780. Upward arrows denote increased IC50 values compared to the parental line. Statistical significance is indicated as follows: ** *p* < 0.01, *** *p* < 0.001.

Cell Line	TOP IC50 (ng/mL)	MIT IC50 (ng/mL)	CIS IC50 (ng/mL)
A2780	9.79 (3.93–17.4) 1.00	2.98 (1.61–4.99) 1.00	11,259 (5719–17,384) 1.00
A2780TR1	200.68 (140.79–241.43) 20.50 ↑**	9.60 (8.52–11.8) 3.22 ↑***	12,213 (7330–17,583) 1.08 ↑
A2780TR2	132.00 (48.27–229.44) 13.48 ↑**	9.65 (8.10–13.7) 3.24 ↑***	12,625 (5528–22,025) 1.12 ↑

**Table 2:** Overview of cell line responses to TOP treatment under 2D culture conditions, both with and without elacridar at concentrations of 0.1, 1, 2, and 5 μM, determined by MTT test. The mean IC50 values for TOP are reported for each cell line, with the range presented in brackets. For reference, cells treated with TOP alone were assigned a relative resistance value of 1. Underlined values represent fold changes in sensitivity in the presence of elacridar, relative to TOP-only treatment. Arrows indicate changes in IC50 values: upward for increases and downward for decreases compared to the control. Statistical significance: ** *p* < 0.01, *** *p* < 0.001.

Cell Line	Control TOP IC50 (ng/mL)	Elacridar 0.1 μM TOP IC50 (ng/mL)	Elacridar 1 μM TOP IC50 (ng/mL)	Elacridar 2 μM TOP IC50 (ng/mL)	Elacridar 5 μM TOP IC50 (ng/mL)
A2780	9.79 (3.93–17.43) 1.00	6.69 (4.53–18.49) 1.46 ↓	10.69 (4.85–17.28) 1.09 ↑	11.18 (5.21–15.3) 1.14 ↑	8.32 (5.19–9.77) 1.17 ↓
A2780TR1	204.68 (140.79–241.43) 1.00	18.81 (13.64–22.04) 10.88 ↓***	15.29 (8.27–21.4) 13.38 ↓***	14.99 (8.34–18.4) 13.65 ↓***	12.05 (4.86–18.85) 16.98 ↓***
A2780TR2	132.00 (48.27–229.44) 1.00	19.11 (9.29–35.64) 6.91 ↓**	11.11 (6.78–15.54) 11.88 ↓***	10.02 (6.03–15.05) 13.17 ↓***	7.61 (6.23–9.52) 17.59 ↓***

**Table 3:** Overview of cell line responses to MIT treatment under 2D culture conditions, both with and without elacridar at concentrations of 0.1, 1, 2, and 5 μM, determined by MTT test. The mean IC50 values for MIT are reported for each cell line, with the range presented in brackets. For reference, cells treated with MIT alone were assigned a relative resistance value of 1. Underlined values represent fold changes in sensitivity in the presence of elacridar, relative to MIT-only treatment. Arrows indicate changes in IC50 values: upward for increases and downward for decreases compared to the control. Statistical significance: * *p* < 0.05, ** *p* < 0.01, *** *p* < 0.001.

Cell Line	Control MIT IC50 (ng/mL)	Elacridar 0.1 μM MIT IC50 (ng/mL)	Elacridar 1 μM MIT IC50 (ng/mL)	Elacridar 2 μM MIT IC50 (ng/mL)	Elacridar 5 μM MIT IC50 (ng/mL)
A2780	2.98 (1.61–4.99) 1.00	2.56 (1.80–3.57) 1.16 ↓	3.08 (2.07–4.22) 1.03 ↑	2.54 (0.91–4.20) 1.17 ↑	2.55 (0.95–4.32) 1.17 ↑
A2780TR1	9.60 (8.52–11.8) 1.00	6.73 (5.20–8.12) 1.43 ↓*	5.25 (4.09–6.29) 1.85 ↓***	2.44 (0.90–4.48) 3.93 ↓***	2.71 (0.93–4.81) 3.55 ↓***
A2780TR2	9.65 (8.10–13.7) 1.00	7.32 (6.48–8.19) 1.32 ↓**	7.40 (6.08–8.39) 1.30 ↓*	5.10 (0.98–8.19) 1.90 ↓*	5.00 (1.37–8.32) 1.93 ↓*

**Table 4:** Overview of cell line responses to CIS treatment under 2D culture conditions, both with and without elacridar at concentrations of 0.1, 1, 2, and 5 μM. The IC50 values for CIS are reported for each cell line. For reference, cells treated with CIS alone were assigned a relative resistance value of 1. Underlined values represent fold changes in sensitivity in the presence of elacridar, relative to CIS-only treatment. Arrows indicate changes in IC50 values: upward for increases and downward for decreases compared to the control.

Cell Line	Control CIS IC50 (ng/mL)	Elacridar 0.1 μM CIS IC50 (ng/mL)	Elacridar 1 μM CIS IC50 (ng/mL)	Elacridar 2 μM CIS IC50 (ng/mL)	Elacridar 5 μM CIS IC50 (ng/mL)
A2780	11,259 (5719–17,384) 1.00	9228 (5174–16,051) 1.22 ↓	9470 (6001–14,572) 1.19 ↓	11,494 (6232–16,100) 1.02 ↑	11,089 (6489–14,751) 1.01 ↓
A2780TR1	12,213 (7330–17,583) 1.00	12,599 (8247–19,394) 1.03 ↑	11,430 (6418–19,332) 1.06 ↓	16,123 (8281–21,191) 1.32 ↑	13,686 (10,139–16,732) 1.12 ↑
A2780TR2	12,625 (5528–22,025) 1.00	13,744 (7392–18,169) 1.08 ↑	13,443 (6763–18,048) 1.08 ↑	11,971 (5840–15,251) 1.06 ↓	9045 (5325–11,274) 1.39 ↓

**Table 5:** Overview of cell line responses to TOP treatment under 3D culture conditions, both with and without elacridar at concentrations of 1 and 5 μM. The IC50 values for TOP are reported for each cell line. For reference, cells treated with TOP alone were assigned a relative resistance value of 1. Underlined values represent fold changes in sensitivity in the presence of elacridar, relative to TOP-only treatment. Arrows indicate changes in IC50 values: upward for increases and downward for decreases compared to the control. Statistical significance: ** *p* < 0.01.

Cell Line	Control TOP IC50 (ng/mL)	Elacridar 1 μM TOP IC50 (ng/mL)	Elacridar 5 μM TOP IC50 (ng/mL)
A2780	17.61 (7.46–32.48) 1.00	11.71 (7.92–18.93) 1.49 ↓	10.19 (8.00–12.22) 1.73 ↓
A2780TR1	447.80 (159.01–774.23) 1.00	15.10 (9.26–20.36) 29.66 ↓**	9.27 (8.70–9.97) 48.31 ↓**
A2780TR2	404.38 (237.89–601.68) 1.00	62.63 (22.30–155.11) 6.46 ↓**	16.02 (8.46–29.91) 25.24 ↓**

**Table 6:** Sequences of specific primers.

Transcript	Sequence (5′–3′ Direction) Forward	Sequence (5′–3′ Direction) Reverse	ENST Number http://www.ensembl.org	Product Size (bp)
BCRP	TTCGGCTTGCAACAACTATG	TCCAGACACACCACGGATAA	00000237612	128
GAPDH	GAAGGTGAAGGTCGGAGTCA	GACAAGCTTCCCGTTCTCAG	00000229239	199

## Data Availability

The data presented in this study are openly available in [https://repod.icm.edu.pl/dataset.xhtml?persistentId=doi:10.18150/G5CYS9 accessed on 16 June 2025] at [doi:10.18150/RITKOQ].

## Abbreviations

The following abbreviations are used in this manuscript:

ABC	ATP-Binding Cassette
ALS	Amyotrophic Lateral Sclerosis
AML	Acute Myeloid Leukemia
BCRP	Breast Cancer Resistance Protein
CIS	Cisplatin
CML	Chronic Myeloid Leukemia
CNS	Central Nervous System
cDNA	Complementary DNA
DOX	Doxorubicin
DPBS	Dulbecco’s Phosphate-Buffered Saline
ECM	Extracellular Matrix
EOC	Epithelial Ovarian Cancer
FDA	Food and Drug Administration
H33342	Hoechst 33342
HCC	Hepatocellular Carcinoma
MIT	Mitoxantrone
MDR	Multidrug Resistance
MSD	Membrane-Spanning Domain
NBD	Nucleotide-Binding Domain
P-gp	P-glycoprotein
PAC	Paclitaxel
PBS	Phosphate-Buffered Saline
PhIP	2-Amino-1-methyl-6-phenylimidazo[4,5-b]pyridine
RNA	Ribonucleic Acid
RT	Reverse Transcription
SCLC	Small Cell Lung Cancer
TM	Transmembrane
TOP	Topotecan
